# The Metabolome Characteristics of Aerobic Endurance Development in Adolescent Male Rowers Using Polarized and Threshold Model: An Original Research

**DOI:** 10.3390/metabo15010017

**Published:** 2025-01-04

**Authors:** Fanming Kong, Miaomiao Zhu, Xinliang Pan, Li Zhao, Sanjun Yang, Jinyuan Zhuo, Cheng Peng, Dongkai Li, Jing Mi

**Affiliations:** 1 Sports Teaching and Research Department, China University of Mining and Technology-Beijing, Beijing 100083, China; 202413@cumtb.edu.cn (F.K.);; 2 Sport Coaching College, Beijing Sport University, Beijing 100084, China; 3 Institute for Emergency Rescue Ergonomics and Protection, China University of Mining and Technology-Beijing, Beijing 100083, China; 4 Liaocheng No. 1 Experimental School, Liaocheng 252001, China; bsuzhumiaomiao@126.com; 5 School of Physical Education, Xi’an University of Architecture and Technology, Xi’an 710055, China; 6 Sport Science School, Beijing Sport University, Beijing 100084, China; 7 Physical Education Department, Renmin University of China, Beijing 100872, China; zhuojinyuan@ruc.edu.cn

**Keywords:** aerobic endurance, polarized training model, threshold training model, rowing, metabolomics

## Abstract

Objective: This study aimed to explore the molecular response mechanisms of differential blood metabolites before and after 8 weeks of threshold and polarized training models using metabolomics technology combined with changes in athletic performance. Methods: Twenty-four male rowers aged 14–16 were randomly divided into a THR group and a POL group (12 participants each). The THR group followed a threshold training model (72%, 24%, and 4% of training time in low-, moderate-, and high-intensity zones, respectively), while the POL group followed a polarized training model (78%, 8%, and 14% training-intensity distribution). Both groups underwent an 8-week training program. Aerobic endurance changes were assessed using a 2 km maximal rowing performance test, and untargeted metabolome analysis was conducted to examine blood metabolomic changes before and after the different training interventions. Aerobic endurance changes were assessed through a 2 km maximal rowing test. Non-targeted metabolomics analysis was employed to evaluate changes in blood metabolome profiles before and after the different training interventions. Results: After 8 weeks of training, both the THR and POL groups exhibited significant improvements in 2 km maximal rowing performance (*p* < 0.05), with no significant differences between the groups. The THR and POL groups had 46 shared differential metabolites before and after the intervention, primarily enriched in sphingolipid metabolism, glutathione metabolism, and glycine, serine, and threonine metabolism pathways. Nine unique differential metabolites were identified in the THR group, mainly enriched in pyruvate metabolism, glycine, serine, and threonine metabolism, glutathione metabolism, and sphingolipid metabolism. A total of 14 unique differential metabolites were identified in the POL group, predominantly enriched in sphingolipid metabolism, glycine, serine, and threonine metabolism, aminoacyl-tRNA biosynthesis, and glutathione metabolism. Conclusions: The 8-week THR and POL training models demonstrated similar effects on enhancing aerobic performance in adolescent male rowers, indicating that both training modalities share similar blood metabolic mechanisms for improving aerobic endurance. Furthermore, both the THR group and the POL group exhibited numerous shared metabolites and some differential metabolites, suggesting that the two endurance training models share common pathways but also have distinct aspects in enhancing aerobic endurance.

## 1. Introduction

Metabolomics, as an effective tool for the quantitative and qualitative analysis of endogenous metabolites, operates downstream of gene regulatory and protein interaction networks, providing terminal biological insights. This offers a new perspective for investigating the intrinsic mechanisms of physical exercise [1,2]. Metabolomics refers to the systematic study of all metabolites within a biological sample using mass spectrometry or nuclear magnetic resonance spectroscopy, with a focus on interactions among various systems within the organism [3,4,5]. It allows for the screening of differential metabolites between individuals and populations while also elucidating the biological mechanisms underlying these differences. With these advantages, metabolomics has emerged as a research hot spot for explaining metabolic changes induced by endurance training [6,7].

Endurance athletes often adopt different intensity distribution models for training, with the threshold training model (THR) and polarized training model (POL) being classic and commonly employed approaches in the field of endurance training. These models are well-supported by theoretical research and have been widely recognized in practical training contexts [8,9]. The POL model’s training intensity distribution (TID) focuses primarily on low-intensity (zone 1, Z1) and high-intensity (zone 3, Z3) sessions, whereas the THR model’s TID emphasizes Z1 and moderate-intensity sessions (zone 2, Z2). Despite the high proportion of Z1 training in both models, the distinct ratios of Z2 and Z3 lead to different energy metabolism pathways and varying stress responses in the cardiovascular system and skeletal muscles, which may result in differences in blood metabolic pathways [10].

Existing studies on training intensity distribution have largely focused on performance outcomes such as time trials, peak power output, and average power in sports like cross-country skiing [11] and cycling [12]. However, metabolomics studies evaluating and monitoring endurance athletes’ physiological states through energy metabolism stages, material transport pathways, and signal transduction mechanisms remain limited [6]. Additionally, factors such as athletic proficiency, training phases, and load measurement standards introduce subjective and objective variations, leaving the effectiveness of these two endurance training models a topic of ongoing debate within academia [13,14,15,16]. Given this context, this study selected adolescent male rowers as experimental subjects and applied metabolomics technology combined with changes in athletic performance to explore the molecular mechanisms underlying the development of aerobic endurance before and after 8 weeks of THR and POL training. This aims to provide scientific evidence for enhancing the efficiency and quality of endurance training in adolescent athletes.

## 2. Materials and Methods

### 2.1. Participants and Group

This study involved 24 adolescent male rowers from a sports school in Shandong Province as experimental subjects, who were randomly divided into the THR group and the POL group, with 12 participants in each group. During the study period, all participants ate and lived at the sports school under consistent conditions, with strict restrictions on the use of mobile phones and other electronic devices. The intake of protein supplements, nutritional supplements, and sports drinks was also prohibited. Detailed information on the participants is provided in Table 1.

### 2.2. Study Design

#### 2.2.1. Experimental Intervention

**Figure 1:**
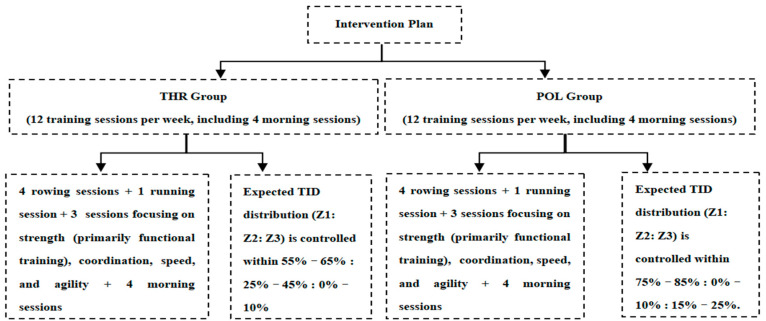
8-week experimental intervention plan.

The THR and POL groups followed similar exercise formats, but their training intensity distribution (TID) differed. Each group participated in 12 training sessions per week, with details shown in Figure 1.

#### 2.2.2. Training Intensity Zones and Load Monitoring

Based on the research of Matzka [17], Sylta [18], and others, this study defined the aerobic threshold (Zone 1, Z1) as blood lactate levels below 2.5 mmol/L, marked as the first lactate threshold (LT_1_). Blood lactate levels between 2.5 and 4 mmol/L were designated as the transition between aerobic and anaerobic zones (Zone 2, Z2), defined as LT_1_ to LT_2_. Blood lactate levels of ≥4 mmol/L were categorized as above the anaerobic threshold (Zone 3, Z3), marked as the second lactate threshold (LT_2_) [13,19]. Heart rate values corresponding to LT_1_ and LT_2_ were calculated using the Lactate Analysis Plugin (LAP) [20]. Polar watches (Polar Pacer, Finland) and the Firstbeat Training and Recovery Monitoring System (Firstbeat, Finland) were used to monitor TID continuously. The Time-in-Zone (TIZ) method was employed to calculate TID, focusing solely on heart rate data from endurance activities such as running and rowing on ergometers. Strength training, speed training, coordination exercises, warm-ups, and recovery sessions were identical in both groups and excluded from the TID calculations. To enhance comparability, the experimental design was modeled on studies by Treff et al. [13] and Chiang et al. [21], ensuring relative equivalence in average time spent, exercise distance, energy expenditure, and Training Impulse (TRIMP) between the three intensity zones for both groups [22,23].

#### 2.2.3. Training Load Calculation Standards

Considering that the Firstbeat Training and Recovery Monitoring System may experience data loss when monitoring heart rate during outdoor running sessions, potentially leading to an underestimation of training intensity distribution, this study used Polar Pacer heart rate data as the primary reference for TID calculations. To provide a more comprehensive and accurate reflection of athletes’ physiological responses during each training session, all heart rate data from the entirety of each session, including warm-ups and cool-downs, were included in the statistics. If an athlete missed one or more complete training sessions within a week, their data for that week were excluded from the analysis [20].

### 2.3. Testing Protocol

Before and after the 8-week training period, participants underwent a 2 km maximal rowing ergometer test. The detailed testing protocol was as follows: Prior to testing, athletes performed a warm-up consisting of 4 laps of slow jogging (approximately 2 min and 30 s per lap), followed by 5 min of joint mobilization and skeletal muscle activation exercises. Next, they completed one 80% effort sprint over 40 m, two 90% effort sprints over 40 m, and one 100% effort sprint over 10 m. After this, a 10 min sport-specific warm-up at a power output of approximately 200 W was conducted to further mobilize the joints. Following the warm-up, athletes rested for 10 min with access to appropriate hydration. Once ready, the ergometer resistance was set to the appropriate level, and the test commenced upon a unified signal from the coach, with participants giving their maximal effort to complete the 2 km rowing test.

### 2.4. Blood and Urine Indicators

To align with the research objectives, blood samples were collected twice, before and after the 8-week training period. Prior to blood collection, all athletes were instructed to avoid high-intensity physical activity for 48 h. Specifically, at 6:30 a.m., a dedicated nurse from a tertiary hospital collected 5 ml of venous blood from each participant using standard collection tubes. The samples were allowed to clot at room temperature for 1 h, followed by centrifugation at 4 °C at 1500× *g* for 10 min. Serum was then transferred to clean centrifuge tubes, snap-frozen in liquid nitrogen for 30 s, and stored in a −80 °C freezer for subsequent testing [24].

Additionally, to enhance the accuracy of the test results, the following precautions were taken during sampling: Athletes were required to avoid staying up late and refrain from eating or drinking after 10 p.m. the night before the test. To ensure the athletes were in a fully rested state, all participants were instructed to sit or lie down quietly for at least 20 min before blood collection. For consistency, pre-test blood samples were stored at −80 °C and analyzed alongside post-test samples using the same batch of reagents. This study was approved by the Ethics Committee of the Beijing Sport University Exercise Science Laboratory (Approval Number: 2023009H), and all testing and training procedures adhered strictly to the guidelines of the World Medical Association Declaration of Helsinki.

### 2.5. Metabolomics Sequencing and Analysis

Polar metabolites were extracted from 50 µL of serum using 200 µL of ice-cold methanol containing 8 µg of phenylhydrazine for α-keto acid derivatization. The samples were vortexed and incubated at −20 °C for 1 h. Subsequently, samples were incubated at 4 °C at 800× *g* for 15 min. After incubation, samples were centrifuged at 4 °C at 600× *g* for 10 min, and the clean supernatant was transferred to a new tube and dried using a centrifugal evaporator in H_2_O mode. The dried extracts were then reconstituted in 120 µL of 5% acetonitrile aqueous solution, and the supernatant was collected for analysis.

#### 2.5.1. Sample Detection Conditions and Quality Control (QC) Methods

Chromatography conditions: Metabolite separation was performed using an ACQUITY UPLC BEH Amide 1.7 µm, 2.1 × 100 mm column (Waters, Dublin, Ireland) for normal-phase chromatography. An ACQUITY UPLC HSS T3 1.8 µm, 3.0 × 100 mm column (Waters, Dublin, Ireland) was used for reverse-phase chromatography.

#### 2.5.2. Mass Spectrometry Conditions

Analysis was conducted using a 5600 Triple TOF Plus high-resolution tandem mass spectrometer (AB Sciex, Singapore) with an electrospray ionization (ESI) source in negative ion mode at a voltage of −4.5 kV. The evaporator temperature was set to 500 °C, with drying gas (nitrogen) and nebulizer gas (nitrogen) pressures both at 50 psi and a curtain gas (nitrogen) pressure of 35 psi. The scan range was *m*/*z* 60–800. The data acquisition mode for metabolites included TOF full-scan for primary mass spectrometry and Information-Dependent Acquisition (IDA) for secondary mass spectrometry, with a collision energy setting of (−)30 ± 15 eV.

#### 2.5.3. Quality Control (QC) Methods

QC samples were prepared by pooling equal volumes (10 µL) of all individual samples, followed by even distribution into aliquots. QC samples were run before and after the main sample queue and at intervals of every 10 samples. The ionization signal of internal standards in QC samples was monitored to ensure stability (within a 20% variation limit) and retention time consistency (within 0.05 min). An average correlation coefficient of 0.99 confirmed high data consistency and quality in LC-MS analyses.

### 2.6. Data Analysis

All data were analyzed using SPSS 20.0 and are presented as mean ± standard deviation (SD). A two-way repeated measures ANOVA was used to compare the changes in the 2 km maximal rowing performance indicators before and after the intervention for the THR and POL groups. A *p*-value of < 0.05 was considered statistically significant.

Data acquisition and processing were conducted using AnalystTF 1.7.1 software (AB Sciex, Concord, ON, Canada). MarkerView 1.3 (AB Sciex, Concord, ON, Canada) was employed to extract peak area, mass-to-charge ratio, and retention time from the primary mass spectrometry data, generating a two-dimensional data matrix with isotope peaks filtered. PeakView 2.2 (AB Sciex, Concord, ON, Canada) was utilized to extract MS/MS data and annotate ion information through comparisons with the Metabolites database, HMDBMETLIN, and reference standards, identifying metabolite IDs. The identified IDs were matched to corresponding ions in the primary mass spectrometry matrix. Custom R-based scripts were applied for statistical and pathway analysis. The peak areas of endogenous metabolites were normalized to those of structurally similar isotope-labeled standards for quantification. For endogenous metabolites without isotope-labeled counterparts, the optimal internal standard was chosen using an automated algorithm based on the normalized minimum coefficient of variation [25]. Differential metabolites were screened using paired sample *t*-tests (*p* < 0.05), and those with fold change (FC) < 1/1.5 or FC > 1.5 were considered significant [26]. Differential metabolites were subjected to Kyoto Encyclopedia of Genes and Genomes (KEGG) pathway enrichment and topology analysis, with significant enrichment determined by *p* < 0.05. Perform a two-factor repeated measures ANOVA to compare the changes in endurance performance indicators before and after intervention between the THR group and the POL group.

## 3. Results

### 3.1. Training Intensity Distribution

Statistical results of training load during the experimental period (Table 2) indicated that the THR group’s training intensity distribution (TID) was 72%:24%:4%, meeting the criteria for the threshold model. The POL group’s TID was 78%:8%:14%, with a polarization index (PI) greater than 2, aligning with the standards of the polarized model [13]. No statistically significant differences were observed between the two groups in terms of total distance, total calorie expenditure, and training impulse (TRIMP), indicating relatively equivalent training loads for both groups.

### 3.2. The 2 km Maximal Rowing Performance

**Figure 2:**
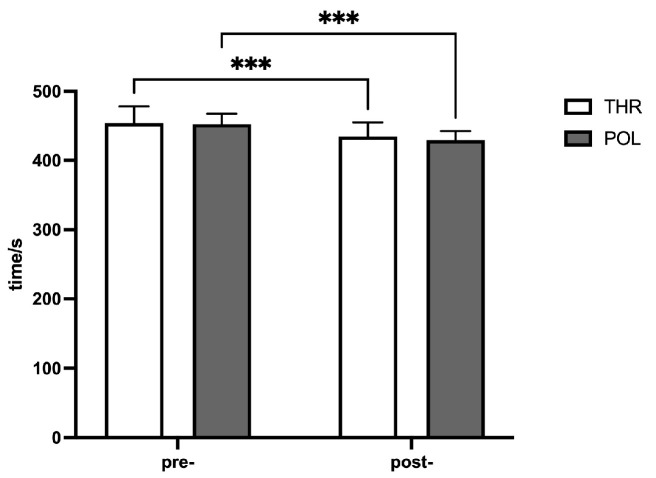
Changes in 2 km maximal rowing performance. Compared to before the 8-week intervention, *** *p* < 0.0001.

Results from two-way repeated measures ANOVA indicated that after the 8-week training intervention, there was no significant interaction effect between time and group for the 2 km ergometer test (F = 1.620, *p* = 0.216). However, the main effect of time was significant (F = 172.0, *p* < 0.0001), while the main effect of the group was not significant (F = 0.2055 *p* = 0.6548). Post-hoc paired comparisons revealed that both groups showed a gradual and significant improvement in their 2 km maximal rowing performance after the 8-week training (*p* < 0.001), with post-test performance significantly better than pre-test performance (*p* < 0.001), with details shown in Figure 2.

### 3.3. Quality Control Results for Metabolomics

**Figure 3:**
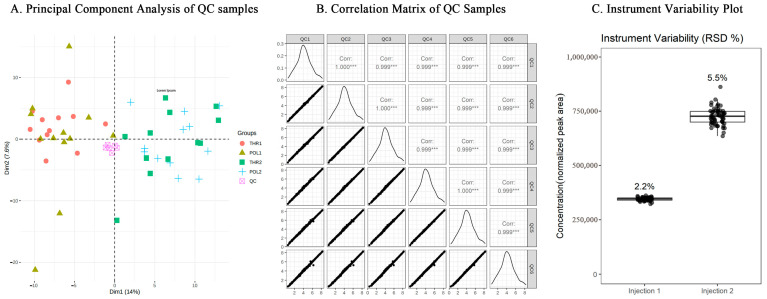
Quality control results of metabolomics experiment data. Note: THR1 denotes the pre-test for the threshold training group, THR2 represents the post-test for the threshold training group, POL1 denotes the pre-test for the polarized training group, and POL2 represents the post-test for the polarized training group; this notation applies throughout (*** represents a high degree of consistency between the data in the two QC samples, reflecting high data quality).

As shown in Figure 3A, the principal component (PC) score plot, including QC samples, reveals a dense clustering of QC samples, indicating stable signals throughout the detection process and high data quality. The data from six QC samples were log10-transformed, and pairwise comparisons were conducted to produce a QC sample correlation matrix. In Figure 3B, the tight linear distribution of all scatter points demonstrates a high degree of consistency between the two QC samples, with corresponding correlation coefficients exceeding 0.99, indicating strong data consistency and high quality. Figure 3C shows that the relative standard deviations (RSD) of the internal standard metabolites were all below 10%, indicating stable instrument signals and high data quality during testing.

### 3.4. Principal Component Analysis

**Figure 4:**
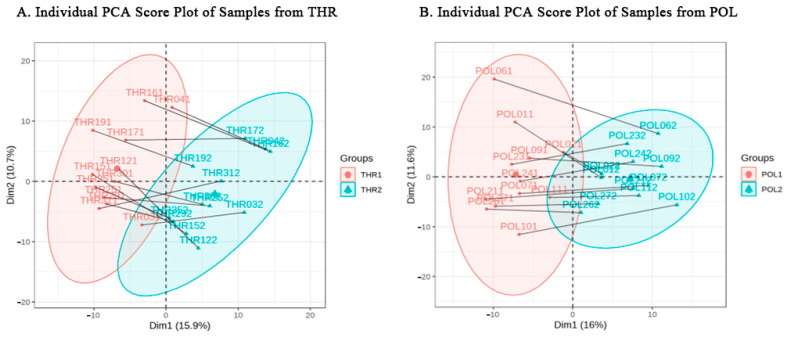
PCA score plot of samples from the THR and POL groups before and after intervention.

As shown in Figure 4A,B, the sample scatter plots for the THR and POL groups during the pre- and post-8-week training intervention at rest exhibit a clear separation trend along the first principal component (PC1) and second principal component (PC2). These results indicate substantial biological metabolic differences in both groups before and after the training intervention.

### 3.5. Differential Metabolite Analysis

**Figure 5:**
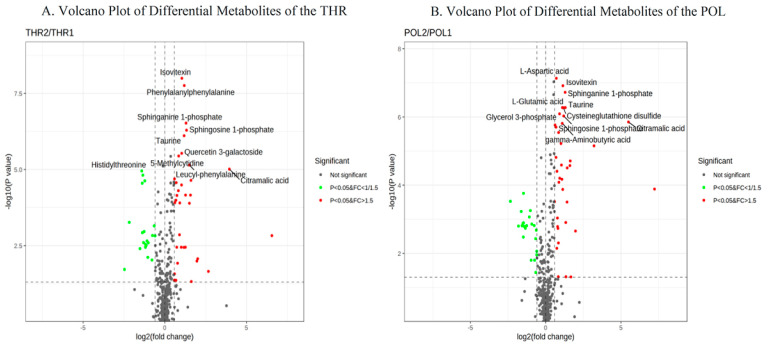
Volcano Plot of Differential metabolites for the THR and POL groups before and after intervention.

The differential metabolite analysis results for the THR and POL groups are detailed in Figure 5A,B. Metabolites were selected based on criteria of *p* < 0.05 and FC > 1.5, or *p* < 0.05 and FC < 1/1.5 [26]. Compared to pre-intervention levels, the THR group exhibited a total of 35 significantly upregulated metabolites after the 8-week training intervention. The top five upregulated metabolites were 12S-HHT, citric acid, oxidized glutathione, dihydromorphine-6-glucuronide, and daidzein, with respective fold changes of 93.62, 15.48, 6.38, 4.03, and 3.92. The POL group, after 8 weeks of training intervention, showed 38 significantly upregulated metabolites, with the top five being 12S-HHT, citric acid, oxidized glutathione, daidzein, and genistein, with fold changes of 150.23, 45.23, 9.26, 3.95, and 3.19, respectively. The THR group also demonstrated 20 significantly downregulated metabolites after the 8-week intervention. The top five downregulated metabolites were sucrose, arachidonic acid, 12-methyltridecanoic acid, histidylserine, and an isomer of mimosine, with fold decreases of 5.51, 4.50, 2.86, 2.65, and 2.60, respectively. The POL group exhibited 22 significantly downregulated metabolites, with the top 5 being arachidonic acid, 12-methyltridecanoic acid, galactose, L-tryptophan, and L-serine, with respective fold decreases of 5.10, 3.50, 3.10, 2.97, and 2.86.

### 3.6. Differential Metabolic Pathway Enrichment Analysis

**Figure 6:**
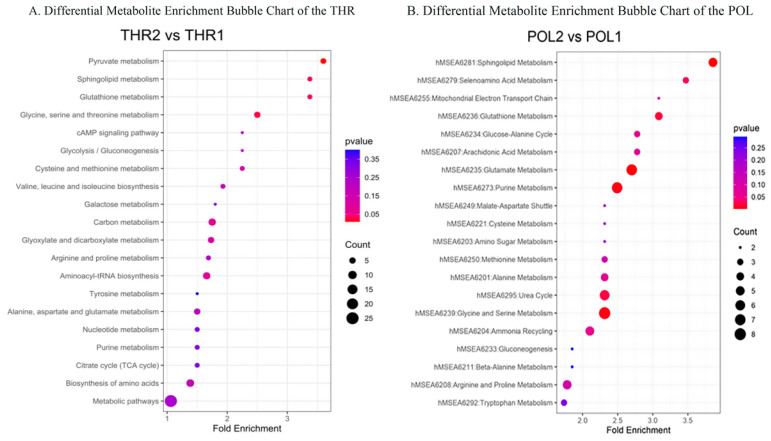
Bubble plot of metabolic pathway enrichment analysis of differential metabolites in the THR and POL groups before and after intervention. Note: Each bubble in the bubble plot represents a metabolic pathway. The size and position of the bubble along the horizontal axis indicate the pathway’s impact factor in the topological analysis, with larger values representing greater impact. The color and position of the bubble along the vertical axis represent the enrichment analysis *p*-value (expressed as the negative natural logarithm, −log(*p*)). Darker bubble colors indicate smaller *p*-values, signifying a higher degree of enrichment.

The differential metabolic pathway analysis results for the THR and POL groups are illustrated in Figure 6A,B. Pathway enrichment analysis of the THR group’s differential metabolites after the 8-week training intervention revealed four significantly enriched pathways: pyruvate metabolism, glycine, serine, and threonine metabolism, glutathione metabolism, and sphingolipid metabolism. Similarly, analysis of the POL group’s differential metabolites identified four significantly enriched pathways: sphingolipid metabolism, glycine, serine, and threonine metabolism, aminoacyl-tRNA biosynthesis, and glutathione metabolism. Detailed results are provided in Table 3.

### 3.7. Analysis of Shared Differential Metabolites

**Figure 7:**
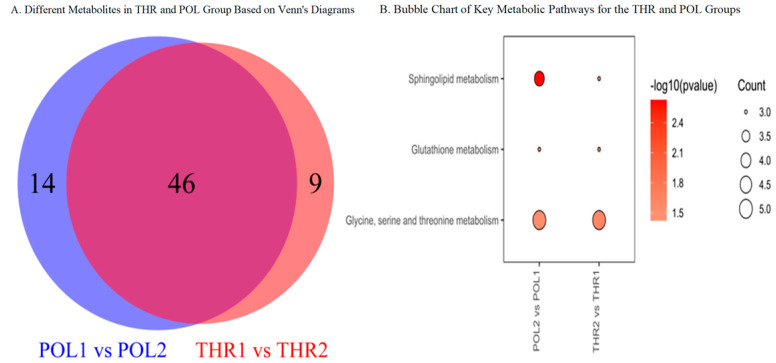
Venn diagram of differential metabolic pathways and bubble plot of key metabolic pathways for the THR and POL groups.

As shown in Figure 7A, after the 8-week training intervention, 46 differentially expressed metabolites were found to be shared between the THR and POL groups. Additionally, the THR group exhibited nine uniquely expressed differential metabolites, while the POL group had 14 uniquely expressed differential metabolites. As illustrated in Figure 7B, the key metabolic pathways commonly enriched for both the THR and POL groups before and after the 8-week intervention include sphingolipid metabolism, glutathione metabolism, and glycine, serine, and threonine metabolism.

## 4. Discussion

### 4.1. Aerobic Endurance Performance

This study found no significant differences in pre-test 2 km maximal rowing performance between the THR and POL groups, indicating comparable baseline levels of sport-specific aerobic endurance. After an 8-week intervention, both the THR and POL groups showed significant improvements in their 2 km ergometer rowing performance, with no significant differences between the groups, suggesting that both training models effectively enhance aerobic endurance performance in adolescent athletes, with comparable effects on aerobic performance. These findings align with the results of Neal [12] and Treff et al. [13]. Neal et al. observed similar outcomes in cycling, where 12 elite male cyclists completed six weeks of either THR or POL training in a randomized crossover design with a four-week washout period between protocols. Both training models effectively increased average power output over a 40 km trial, with similar improvements between groups. Treff et al. [13] examined 14 elite male rowers (aged 20 ± 2 years) over 11 weeks of THR and POL training and found comparable improvements in 2 km rowing performance.

### 4.2. Shared Metabolites Enhancing Aerobic Capacity

Results demonstrated significant changes in blood metabolite profiles following 8 weeks of THR and POL training in adolescent male rowers. Endurance, or fatigue resistance, improvements are often reflected in the ability to maintain specific loads or movement quality for longer periods, with increased anti-fatigue capacity or reduced recovery times serving as important indicators of enhanced endurance. Exercise requires coordinated responses from the cardiovascular and respiratory systems to minimize stress on metabolic organs and meet increased energy demands [5]. Improvements in aerobic capacity represent a chronic adaptation involving cardiovascular, respiratory, skeletal muscle, metabolism, and body composition changes. This adaptation encompasses energy metabolism, fatigue and recovery, oxidative stress response, neurometabolism, amino acid metabolism, cell damage and repair, and signaling pathways. Consequently, improvements in energy metabolism, fatigue resistance, recovery, antioxidation, nutritional supplementation, anti-aging, and anti-inflammatory processes enhance aerobic endurance.

#### 4.2.1. Regulating Energy Metabolism and Increasing Energy Reserves

The levels of citric acid in both the THR and POL groups increased by 15.48-fold and 45.23-fold, respectively, following 8 weeks of endurance training, indicating a positive correlation with aerobic endurance. This suggests that both high-intensity and moderate-to-low-intensity training stimulate citric acid metabolism, potentially marking it as an indicator of improved aerobic endurance. The citric acid cycle plays a central role in the oxidation of metabolic substrates and ATP production through oxidative phosphorylation. In a normal physiological state, approximately 75% of cardiac ATP is derived from fatty acids, with the remainder from glucose and lactate. Additionally, the heart can utilize pyruvate, amino acids, and ketone bodies as metabolic substrates, primarily through mitochondrial β-oxidation of fatty acids and glycolysis of glucose in the cytoplasm, followed by mitochondrial pyruvate oxidation. These pathways converge to produce acetyl-CoA, which enters the TCA cycle, generating reduced equivalents (NADH+, FADH+) and GTP, which are converted to ATP through the electron transport chain [27]. As the initial compound in the TCA cycle, citric acid converts carbohydrates, fats, and proteins into carbon dioxide, releasing energy [7]. Increased resting levels of citric acid in athletes indicate enhanced aerobic energy contribution and improved mitochondrial TCA cycle function, suggesting enhanced aerobic metabolic activity [28,29].

L-glutamate, L-malic acid, and other amino acids showed significant upregulation, while L-leucine, L-threonine, and L-alanine were significantly downregulated. These metabolites, classified as glucogenic amino acids, can be converted to glucose via gluconeogenesis for energy production. Glycine, another glucogenic amino acid, can be converted to pyruvate, entering the TCA cycle for aerobic oxidation. These changes indicate enhanced glucose metabolism, a hallmark of improved aerobic capacity. This aligns with previous studies, such as Gao Huan et al. [28], who examined metabolic responses in eight adolescent rowers (age: 16.18 ± 1.34 years) following 4 weeks of THR training (TID: 74%:26%:0). Glutamate, glycine, and alanine were identified as key markers of aerobic metabolism. Additionally, increased levels of arachidonic acid, a precursor for endocannabinoid synthesis, activate endocannabinoid signaling, modulating metabolism to accumulate energy reserves, reduce glucose utilization in skeletal muscle, and promote hepatic fat synthesis, thereby reflecting enhanced exercise capacity [30]. 

#### 4.2.2. Reduce Oxidative Stress and Improve Metabolic Environment

Intense or prolonged exercise accelerates substance and energy metabolism, increasing free radical levels. Elevated reactive oxygen species stimulate vagal afferent fibers in the ventricles, reducing aerobic endurance. Glutathione and taurine effectively scavenge intracellular free radicals, reducing oxidative stress and mitigating lipid peroxidation [31]. Taurine supplementation enhances myocardial superoxide dismutase activity, increasing myocardial contractility and promoting post-exercise heart rate recovery [31]. Enhanced free radical scavenging improves the body’s internal environment and, consequently, aerobic capacity.

This study showed significant upregulation of 12S-HHT in both the THR and POL groups following 8 weeks of endurance training, with increases of 93.62-fold and 150.23-fold, respectively, potentially serving as a new target for evaluating aerobic endurance improvement. 12S-HHT, an enzymatic product of prostaglandin H2, originates from COX-mediated arachidonic acid metabolism and exhibits protective cellular effects, including nitric oxide regulation, cellular morphology maintenance, and cell flow regulation, enhancing immunity, anti-inflammatory, and antioxidant functions [32].

Daidzein and genistein also exhibit cardioprotective effects. Daidzein reduces arterial sclerosis, mitigates hypoxia and arrhythmia, and modestly lowers blood pressure, benefiting cardiovascular health. Mechanisms by which daidzein improves aerobic capacity may include coronary artery dilation, reduced cardiovascular resistance, and lower myocardial oxygen consumption. Daidzein also reduces thromboxane A2 (TXA2) and prostaglandin-I-2 (PGI2), inhibiting platelet aggregation, reducing blood viscosity, and improving microcirculation. Similarly, genistein, a flavonoid, mitigates arsenic trioxide-induced cardiac toxicity, suppresses endothelial cell inflammation, modulates lipid metabolism, and prevents cardiac fibrosis. Its protective mechanisms may involve inhibiting mitochondrial apoptosis proteins, activating Akt (protein kinase B) and ERK1/2 (extracellular signal-regulated kinases), and enhancing protein expression [33].

#### 4.2.3. Promoting Erythropoiesis and Enhancing Fatigue Resistance

Erythrocytes, the most abundant blood cells, serve as crucial oxygen carriers. Their number is an essential indicator of aerobic capacity. Taurine, oxidized glutathione, and 12S-HHT may play key roles in promoting erythropoiesis. Under fatigue or overtraining, erythrocyte morphology may be altered, while taurine enhances iron absorption in the gut, stabilizes erythrocyte membranes, and improves cell structure [31]. Oxidized glutathione regulates aerobic capacity by converting to reduced glutathione via glutathione reductase, preventing hemoglobin oxidation and reducing erythrocyte oxidative damage. Additionally, Okuno et al. [34] found that 12S-HHT is associated with cytochrome P450 enzymes regulating erythrocyte synthesis, and its upregulation promotes erythropoiesis, contributing to improved aerobic endurance. Quintas et al. [35] revealed significant changes in γ-aminobutyric acid (GABA) levels in elite female soccer players over a season using urine metabolomics. As the primary inhibitory neurotransmitter in the central nervous system, GABA plays a crucial role in balancing neuronal excitation and inhibition, promoting sleep and fatigue resistance.

### 4.3. Differential Metabolites Enhancing Aerobic Capacity

As described above, after the 8-week endurance training intervention, the THR group uniquely expressed nine metabolites, including dihydromorphinone-6-glucuronide, 4-vinylphenol sulfate, hydroxycaprylic acid, L-malic acid, 4-ethylphenyl sulfate, mimetic acid, 7,8-dihydrobiopterin, heptadecanoic acid, and sucrose. Pathway analysis showed that the THR group’s unique metabolites were mainly enriched in pyruvate metabolism, which is closely related to the tricarboxylic acid (TCA) cycle. This suggests that the THR training model improves aerobic capacity primarily through enhancing energy metabolism.

Similarly, the POL group uniquely expressed a total of 14 metabolites after the 8-week endurance training intervention, including genistein, O-phosphoethanolamine, 3-indolepropionic acid, unknown sulfated steroids, hydroquinone sulfate, L-aspartic acid, C16 sphingosine-1-phosphate, β-citryl-L-glutamate, butyric acid, allantoin, glutaric acid, 4-(but-2-yl)cyclohexane-1-carboxylic acid, valproic acid, and 2-aminoheptanoic acid. Pathway analysis revealed that the POL group’s unique metabolites were enriched in aminoacyl-tRNA biosynthesis, a pathway associated with skeletal muscle protein synthesis. This molecular response perspective further suggests that the POL model’s improvement of aerobic capacity is more inclined towards peripheral mechanisms, which may be related to the body’s differential activation of energy metabolism systems under varying intensity stimuli [10].

In summary, both the THR group and the POL group have both shared and differential metabolites, with the number of common metabolites significantly outnumbering the differential ones. This suggests that the two endurance training models share common pathways but also have their respective unique aspects in enhancing aerobic endurance.

### 4.4. Limitations

Although this study identified potential metabolic targets for reflecting aerobic capacity, further validation through larger sample sizes is needed. Future research should focus on targeted metabolomics for key metabolic organs such as the heart and skeletal muscle to enhance detection specificity and provide more scientifically robust evidence. Given the predominance of aerobic endurance data in cardiovascular disease-related databases, future studies should emphasize systematic integration and correlation analysis of similar or functionally related metabolites to advance understanding in this domain.

## 5. Conclusions

After 8 weeks of training using the THR model (TID of 72%:24%:4%) and the POL model (TID of 78%:8%:14%), the two groups exhibited a high number of commonly expressed metabolites and shared enriched pathways. This indicates that both training models enhance aerobic endurance through similar physiological mechanisms, which may be attributed to a comparable proportion of Z1 intensity, relatively equivalent training loads, and a high overall training volume in both experimental groups. Furthermore, both the THR group and the POL group exhibited numerous commonly expressed metabolites and some differential metabolites, suggesting that the two endurance training models share common pathways but also have distinct aspects in enhancing aerobic endurance.

The mechanisms by which the commonly expressed metabolites enhance aerobic capacity primarily involve regulating energy metabolism, increasing energy reserves, reducing oxidative stress, protecting myocardial cells, promoting erythropoiesis, and improving fatigue resistance. 

Our study, for the first time, identified metabolic biomarkers in adolescent rowing athletes after lactate threshold training and polarized training, providing a new theoretical foundation and experimental evidence for revealing the biological mechanisms underlying the enhancement of aerobic capacity. Specifically, the study found that certain metabolites in adolescent rowing athletes, which are associated with nutritional supplements, exhibited significant physiological functions and possessed potential as nutritional supplements after an 8-week endurance training program. 

## Figures and Tables

**Table 1:** Basic characteristics of the research subjects (n = 24).

Group	n	Bone Age (Years)	Height (cm)	Weight (kg)	BMI (kg/m^2^)	Body Fat Percentage (%)	Training Years (Years)
THR	12	15.38 ± 1.21	1.79 ± 0.06	71.13 ± 11.76	22.12 ± 2.51	11.52 ± 4.17	1.36 ± 0.61
POL	12	15.68 ± 1.11	1.80 ± 0.05	72.23 ± 7.42	22.39 ± 2.05	13.29 ± 3.55	1.35 ± 0.78

Note: Data are presented as mean ± standard deviation. THR = threshold training model; POL = polarized training model; BMI = Body Mass Index.

**Table 2:** Overview of training load statistics for THR and POL groups.

Group	Total Training Duration (h)	Z1 Training Duration (h)	Z2 Training Duration (h)	Z3 Training Duration (h)	Training Intensity Distribution	Total Distance (km)	Total Calories (kcal)	TRIMP	PI
THR	105.89 ± 7.25	76.22 ± 7.52	25.75 ± 2.49	3.91 ± 3.03	72%:24%:4%	2259.00 ± 261.99	53744.67 ± 3876.16	6775.43 ± 1168.77	—
POL	103.72 ± 11.25	80.78 ± 11.05	8.90 ± 1.95	14.05 ± 2.04	78%:8%:14%	2252.13 ± 129.16	50522.75 ± 6774.66	6578.38 ± 520.12	2.11 ± 0.12

Note: All data were obtained from Polar Flow and represent average values over the 8-week training period. TRIMP denotes training impulse, while PI (polarization index) represents the polarization index. “—” indicates that data for this group is not available.

**Table 3:** Overview of key metabolic pathways and metabolites before and after 8 weeks of training in the THR Group.

No.	Pathway Name	Metabolites	*p*-Value	Enrichment Fold
1	Pyruvate Metabolism	Fumaric acid, L-Lactic acid, L-Malate, Pyruvic acid	0.0085	3.60
2	Glycine, Serine, and Threonine Metabolism	Glycine, L-Serine, L-Threonine, Pyruvic acid, 2-Ketobutyric acid	0.025	2.50
3	Glutathione Metabolism	Glycine, L-Glutamic acid, Oxidized Glutathione	0.034	3.38
4	Sphingolipid Metabolism	L-Serine, Sphingosine 1-phosphate	0.034	3.38

Note: The table lists the metabolic pathways and metabolites that were significantly altered in the Threshold Training (THR) group following eight weeks of training, along with their respective *p*-values and enrichment fold changes.

## Data Availability

Data are contained within the article.
